# Characterization of shed medicinal leech mucus reveals a diverse microbiota

**DOI:** 10.3389/fmicb.2014.00757

**Published:** 2015-01-09

**Authors:** Brittany M. Ott, Allen Rickards, Lauren Gehrke, Rita V. M. Rio

**Affiliations:** Department of Biology, West Virginia UniversityMorgantown, WV, USA

**Keywords:** symbiosis, leech, microbiota, Illumina, 16S rRNA, mucus, transmission

## Abstract

Microbial transmission through mucosal-mediated mechanisms is widespread throughout the animal kingdom. One example of this occurs with *Hirudo verbana*, the medicinal leech, where host attraction to shed conspecific mucus facilitates horizontal transmission of a predominant gut symbiont, the Gammaproteobacterium *Aeromonas veronii*. However, whether this mucus may harbor other bacteria has not been examined. Here, we characterize the microbiota of shed leech mucus through Illumina deep sequencing of the V3-V4 hypervariable region of the 16S rRNA gene. Additionally, Restriction Fragment Length Polymorphism (RFLP) typing with subsequent Sanger Sequencing of a 16S rRNA gene clone library provided qualitative confirmation of the microbial composition. Phylogenetic analyses of full-length 16S rRNA sequences were performed to examine microbial taxonomic distribution. Analyses using both technologies indicate the dominance of the Bacteroidetes and Proteobacteria phyla within the mucus microbiota. We determined the presence of other previously described leech symbionts, in addition to a number of putative novel leech-associated bacteria. A second predominant gut symbiont, the *Rikenella*-like bacteria, was also identified within mucus and exhibited similar population dynamics to *A. veronii*, suggesting persistence in syntrophy beyond the gut. Interestingly, the most abundant bacterial genus belonged to *Pedobacter*, which includes members capable of producing heparinase, an enzyme that degrades the anticoagulant, heparin. Additionally, bacteria associated with denitrification and sulfate cycling were observed, indicating an abundance of these anions within mucus, likely originating from the leech excretory system. A diverse microbiota harbored within shed mucus has significant potential implications for the evolution of microbiomes, including opportunities for gene transfer and utility in host capture of a diverse group of symbionts.

## Introduction

Host-generated mucus may harbor both pathogenic and beneficial microbes (Rohwer et al., 2002; Sekar et al., 2006; Sharon and Rosenberg, 2008; Krediet et al., 2009; Shnit-Orland and Kushmaro, 2009) with numerous examples of mucus-mediated microbial transmission occurring throughout the Animal kingdom. For instance, the bobtail squid (*Euprymna scolopes*) uses mucosal secretions for the aggregation of its bioluminescent symbiont, *Vibrio fischeri*, from the surrounding water facilitating its migration into the light organ (Nyholm et al., 2000). Representing basal metazoans, the hydra (*Hydra vulgaris*) contains an external mucosal layer, termed the glycocalyx, where critical symbionts are recruited during early host embryogenesis (Fraune et al., 2010). Additionally, the mucosal secretions of humans (i.e., sputum and nasal secretions) provide a protective environment, particularly in terms of humidity and salinity (Thomas et al., 2008), enabling the transmission of infectious respiratory agents between individuals.

The sanguivorous European medicinal leech, *Hirudo verbana* (Hirudinida: Hirudinidae) uses a dual mode of transmission for acquiring a predominant gut symbiont, the Gammaproteobacterium *Aeromonas veronii* (Ott et al., 2014). Vertical transmission of *A. veronii* occurs during cocoon development likely by the albumenotrophic activity of larvae (Rio et al., 2009), while colonization of adults is through contact with shed mucus that contains a proliferating *A. veronii* population that originates from the digestive tract (Ott et al., 2014). Importantly, leeches are attracted to mucus produced by conspecifics (Ott et al., 2014), which facilitates symbiont horizontal spread. This mixed mode of transmission has significant implications for the evolution of symbiosis, including enabling the capture of a more genetically diverse symbiont population with an enhanced ability to adapt to environmental changes. Furthermore, lifestyle options beyond that of mutualism with the leech may be possible for the mucus-inhabiting bacteria.

The shedding of mucus by medicinal leeches occurs every 2–3 days (Ott et al., 2014) always in an anterior to posterior direction. In addition to catalyzing *A. veronii* horizontal transmission, these mucosal casts have been proposed to serve a multitude of other roles ranging from protection against UV rays and desiccation to facilitating conspecific recognition (Michalsen et al., 2007). Within shed mucus, the *A. veronii* population originates from the leech digestive tract with density maximizing just prior to a new secretion (Ott et al., 2014). This suggests the coupling of symbiont population dynamics and host biology, as well as the potential for other microbial inhabitants within shed mucus to provide metabolic and/or structural support enabling *A. veronii* proliferation.

While leech shed mucus was demonstrated to aid in the transmission of a sole gut symbiont, a moderately rich microbiota (~36 taxa) is actually housed within the *H. verbana* GI tract (Maltz et al., 2014). The leech GI tract is primarily composed of two parts; a crop, where the blood meal is stored, and a smaller intestinum, the actual site of nutrient absorption (Sawyer, 1986). In addition to *A. veronii*, a second predominant Bacteroidetes symbiont, a *Rikenella*-like bacterium, is also localized to the leech gut. Within the host GI tract, the *A. veronii* and the *Rikenella*-like symbionts are synergistic (Kikuchi and Graf, 2007) based on glycan utilization (Bomar et al., 2011), and the likelihood that *A. veronii* may reduce the oxygen supply, enabling the habitation of the anaerobic *Rikenella*-like symbiont.

In addition to the gut, a second endogenous microbiota has also been described in the leech excretory system, consisting of multiple pairs of nephridia connected to bladders that lie alongside the lateral caeca of the crop. A maximum of six bacterial species reside within the bladder, with infection rates of these taxa varying between different leech individuals (Kikuchi et al., 2009). Interestingly, symbiont species display a stratified spatial arrangement within the bladder suggesting a community organization likely impacted by resource availability and output metabolism. The functional basis of the excretory tract symbionts may lie in the recycling of carbon and nitrogenous waste (Kikuchi et al., 2009), which may enable the leech host to sustain long gaps, often as long as 6–12 months (Zebe et al., 1986), between blood meals.

In this paper, we characterize the composition and relative abundance of the microbiota within shed leech mucus using Illumina deep sequencing of the V3-V4 hypervariable region of the 16S rRNA gene. Additionally, Restriction Fragment Length Polymorphism (RFLP) typing and Sanger sequencing of a 16S rRNA clone library provided qualitative confirmation. Phylogenetic analyses of full-length 16S rRNA sequences were performed to examine both the microbial diversity and their evolutionary relations within leech shed mucus. Lastly, following the identification of the *Rikenella*-like symbiont within mucus, its population dynamics were compared with the *A. veronii* symbiont (Ott et al., 2014). The discovery of a rich microbial community within mucus suggests its utility for genetic mixing and resource partitioning within this setting. This species assemblage raises questions pertaining to microbial dynamics and whether these other bacteria, as a group, may also utilize mucus as a means for horizontal transmission.

## Materials and methods

### Leech husbandry

Medicinal leeches (*H. verbana*), were obtained from the medical supplier Leeches USA (Westbury, NY, USA), and housed in sterile Leech Strength Instant Ocean H_2_O (0.001% I.O.) at 15°C at constant darkness. Leeches were maintained on defibrinated bovine blood (Hemostat, CA).

### Mucus sampling

Day 3 mucus (i.e., 3 days post shedding) was chosen for both the construction of a mucosal 16S rRNA clone library and Illumina 16S rRNA deep sequencing, as this time point corresponds to the peak of *Aeromonas* population size (Ott et al., 2014). Mucosal samples used for describing the *Rikenella*-like bacterium population dynamics through time were obtained at 1, 3, 5, or 8 d post shedding within sterile Leech Strength I.O. H_2_O. All samples were snap frozen at −80°C until further processing.

### High-throughput amplicon sequencing analyses

The microbial community of shed mucus was characterized using barcoded Illumina sequencing. Total DNA was extracted from shed mucus using the Holmes-Bonner Protocol (Holmes and Bonner, 1973) and tested for purity on a NanoDrop 2000 spectrophotometer (Thermo Scientific, Waltham, MA). This DNA served as template for PCR amplification of the V3-V4 hypervariable region of the 16S rRNA gene (Klindworth et al., 2013) using the V3Met (5′-CCTACGGGAGGCAGCAG-3′) and MetaV4 (5-GGACTACHVGGGTWTCTAAT-3′) primers. Amplicons (1 × 250 bp, paired end) were sequenced on the Illumina MiSeq platform in the West Virginia University Genomics Core Facility following the manufacturer's protocols (Illumina, CA). Sequence quality control was performed using mothur (Kozich et al., 2013) following the MiSeq SOP (http://www.mothur.org/wiki/MiSeq_SOP; date accessed page August 30, 2014). The screen.seqs command was used to trim the sequence when the average quality score over a 50 bp window dropped below 35, and to eliminate any sequences that were not in the 400–500 bp range. The unique.seqs command was used to cluster the sequences that were within 2 bp of similarity to a more abundant sequence. Sequences were aligned to the SILVA-compatible alignment database using align.seqs and then trimmed to a common region (i.e., 6388 to 25316 of *Escherichia coli*) using the filter.seqs command to remove any overhangs. The classify.seqs and remove.lineage commands were utilized to identify and remove mitochondrial, chloroplast, Archaea, Eukarya and unknown contaminants. Bacterial taxonomy was assigned to each sequence in the improved data set using the classify.seqs command, which uses the Naïve Bayesian classifier of RDP (Schloss et al., 2009). Following taxonomic assignment, sequences were assigned to operational taxonomic units (hereafter OTUs) at the 3% level of divergence using the cluster.seqs command. Here, a 16S rRNA sequence is considered derived from a known genus if the read similarity was ≥ 95% (Schloss and Handelsman, 2005). The read counts at each taxonomy level were normalized to total relative abundance.

### Mucosal 16S rRNA clone library

To assess the microbial diversity within leech shed mucus, total DNA was extracted from 3 d old mucosal sheds of two individuals, using the Holmes-Bonner protocol (Holmes and Bonner, 1973), and subjected to PCR using 27F′ and 1492R′ general eubacteria primers (Lane, 1990; Weisburg et al., 1991) (T_*a*_ [annealing temperature] = 50°C; 28 cycles; amplicon ~1450 bp). PCR products were cloned using the pGEM-T Easy Vector cloning kit (Promega, WI), with subsequent transformation into JM109 *Escherichia coli* cells (Promega, WI). Inserts were amplified using M13F′ and M13R′ vector primers (T*_a_* = 46°C; 35 cycles; amplicon ~1636 bp) and digested with *HaeIII* restriction endonuclease (NEB, Ipswich, MA, USA) for RFLP typing. Clones with unique restriction profiles were purified and subject to Sanger sequencing using M13 primers with an ABI Genetic Analyzer 3130*xl* at the WVU Department of Biology Genomics Center. The DNA sequences were aligned and assembled into contigs and identified to the highest taxonomic level possible using nucleotide Basic Local Alignment Search Tool (BLASTn, http://blast.ncbi.nlm.nih.gov/Blast.cgi).

### Molecular phylogenetic analyses

To examine microbial community diversity and their relations, phylogenetic trees including the 16S rRNA sequences that were identified within mucus samples, close relatives, and previously identified *H. verbana* leech isolates (Worthen et al., 2006; Kikuchi et al., 2009) were constructed. DNA sequences were aligned using the Clustal X algorithm with default settings, and refined manually when necessary. Maximum parsimony (MP) analyses were performed with 1000 replicates in PAUP 4.0 (Swofford, 2002). MP heuristic searches utilized the tree-bisection-reconnection (TBR) branch-swapping algorithm with 200 Max trees and starting trees were created using stepwise additions. All MP analyses were performed twice, where gaps were treated either as “missing data” or as a “5th character state,” with no differences noted between the results. Lineage support was measured by calculating nonparametric bootstrap (BS) values (*n* = 1000) (Felsenstein, 1985).

A second tree was produced for the same alignment using the Bayesian Markov Chain Monte Carlo method as implemented with MrBayes (3.1.2) (Ronquist and Huelsenbeck, 2003). The best-fit model (GTR+I+G) used for Bayesian analyses was statistically selected using the Akaike Information Criterion in MrModeltest version 2.3 (Nylander, 2004). Bayesian analyses were performed with six Markov chains (Larget and Simon, 1999) for 5,000,000 generations. Posterior probabilities (PP) were calculated, with the stabilization of the model parameters (i.e., burn-in) occurring around 4,000,000 generations. Every 100th tree following stabilization was sampled to determine a 50% majority rule consensus tree. All trees were made using the program FIGTREE v.1.4.0 (http://tree.bio.ed.ac.uk/software/figtree/).

### *Rikenella*-like bacteria population dynamics

The population dynamics of *Rikenella*-like symbionts within host mucus at 1, 3, 5, and 8 d following secretion were determined by utilizing real-time quantitative PCR (q-PCR). DNA isolation from mucus was performed following a Holmes–Bonner protocol (Holmes and Bonner, 1973). Analyses were performed in an iCycler iQ Real-Time PCR Detection System (Bio-Rad, Hercules, CA, USA) using Bio-Rad SsoFast EvaGreen Supermix, 10 mM of primers *rpoD*F′ q-PCR (5′-AGT TGC GGA CAC TCT ACG TG-3′) and *rpoD*R′ q-PCR (5′-TCC AAG AGC GTG TTG TCT TC-3′) (T_*a*_ = 55°C; 35 cycles; amplicon = 84 bp) and 2 μL of DNA template as described (Rio et al., 2009). Quantification of the amplicons relative to standard curves was performed using Bio-Rad CFX Manager software v 2.0. The respective DNA concentration (ng/ul) of each sample was used for the normalization of copy numbers. All assays were performed in triplicate and replicates were averaged for each sample.

### Statistical analyses

Mean microbial species richness values were obtained using the Shannon-Weaver diversity index (Shannon, 1948). A rarefaction curve was generated using Hurlbert's formulation (Hurlbert, 1971) and vegan version 2.0-10 (Okansen) using the “rarefy” function. The R package “Fossil” was used to generate Chao1, Chao2, ICE, and ACE richness estimates. Percent identities of the16S rRNA sequences between different *Pedobacter* isolates and species were generated using MEGA 6.06 (Tamura et al., 2013) implementing the Jukes-Cantor Model for nucleotide substitution (Jukes and Cantor, 1969). Statistically significant differences in *Rikenella*-like symbiont density within mucus through time were determined by performing a one-way ANOVA using JMP 10 (SAS Institute Inc., Cary, NC, USA) software, with a significance value set at *p* ≤ 0.05.

### Confirmation of reagent purity

The DNA extraction buffer, ultrapure water and PCR kit used for nucleic acid extraction and amplification respectively, were verified to be contaminant-free using PCR amplification with general eubacterial primers.

### Nucleotide sequence accession numbers

The complete sequence dataset for the Illumina reads is available from NCBI under BioProject ID PRJNA269158. Individual sequences generated by the Sanger-sequenced 16S rRNA clone library are available at Genbank under accession numbers KP231731-KP231772.

## Results

### Shed mucus harbors a diverse microbial community

**Figure 1:**
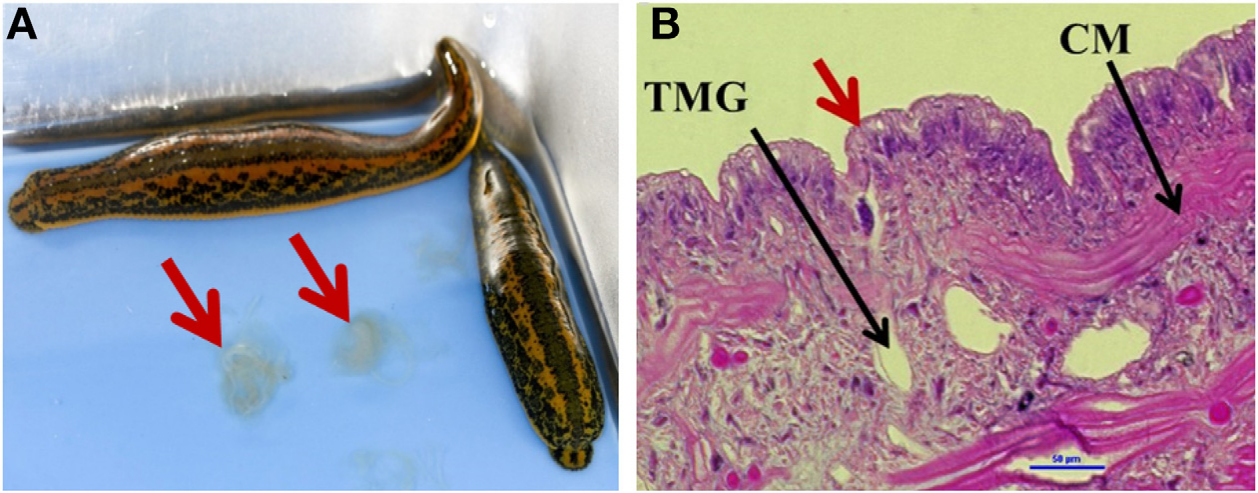
**Medicinal leech mucosal secretions. (A)** *Hirudo verbana* leeches, with arrows indicating shed mucosal secretions. **(B)** The epithelial layer of the leech (20X magnification, Hemotoxylin and eosin (H&E) stain). Red arrow indicates tubular mucus gland cell opening. TMG = tubular mucus gland cell (one of two types of mucus gland cells Sawyer, 1986), CM, circular muscle.

To obtain an understanding of the microbiome profile within shed mucus (Figure 1A), Illumina deep-sequencing of the V3-V4 hypervariable region of the 16S rRNA gene was performed. These mucosal secretions, consisting primarily of glucosaminoglycans, are produced by mucus gland cells that are irregularly distributed beneath the external leech epithelia (Sawyer, 1986; Michalsen et al., 2007) (Figure 1B). Mucus 3 d post shedding was selected for the characterization of the microbiota as this time point corresponds to the peak in density of a predominant leech gut symbiont, *A. veronii* (Ott et al., 2014), and we were particularly interested to know if other bacteria inhabited mucus at this time.

**Figure 2:**
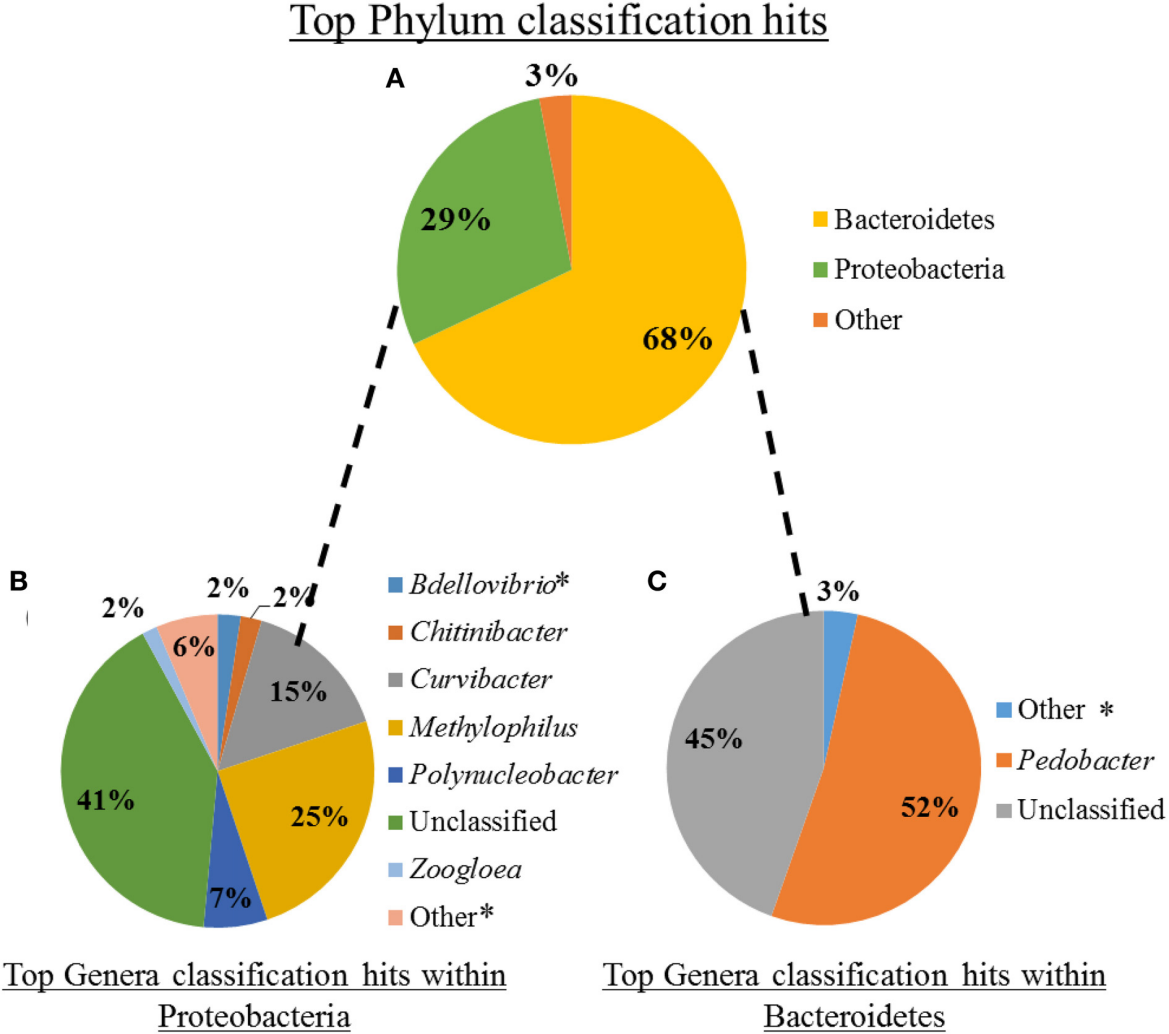
**Microbiota composition of shed leech mucus obtained through Illumina sequencing of the V3-V4 hypervariable region of 16S rRNA gene. (A)** The relative abundance of total reads, following quality control, within phyla. **(B)** The relative abundance of reads, comprising ≥2% of total reads, of genera housed within Proteobacteria. ^*^Indicates previously described within the leech (Worthen et al., 2006; Kikuchi et al., 2009). **(C)** The relative abundance of reads, comprising ≥1% of total reads, of genera housed within Bacteroidetes.

Quality-filtered reads from the mucosal Illumina dataset were assigned to a reference OTU housed in the phylum Bacteroidetes (~68% of reads), Proteobacteria (~29% of reads), or were categorized as “Other” (3% of reads) (Figure 2A). Of these a total of 2,136,157 reads (~98%) could be assigned to the genus level, with an additional 34,784 sequences unclassifiable to this level. Approximately 4% of all reads (84,918 reads) were to genera that constituted <1% of the mucosal microbiota. Genera that contained ≥1% of total reads included *Bdellovibrio* (2%), *Chitinibacter* (2%), *Curvibacter* (15%), *Methylophilus* (25%), *Polynucleobacter* (7%), and *Zoogloea* (2%) within Proteobacteria (Figure 2B) and *Pedobacter* (52%) within Bacteroidetes (Figure 2C). Interestingly, the most prevalent 16S rRNA OTUs obtained within Bacteroidetes are housed in the *Pedobacter* genus (~52% of OTUs), which has never been identified within the medicinal leech. However, *Bdellovibrio* sp. (~2% of OTUs), which has been previously described within the nephridial system, was found within the Proteobacteria phylum (Kikuchi et al., 2009). Additionally, the predominant gut bacteria, *A. veronii* and the *Rikenella*-like bacterium, were also detected, but both consisted of < 1% of total OTU abundance in their respective phyla.

### RFLP typing of the mucosal microbial community

**Figure 3:**
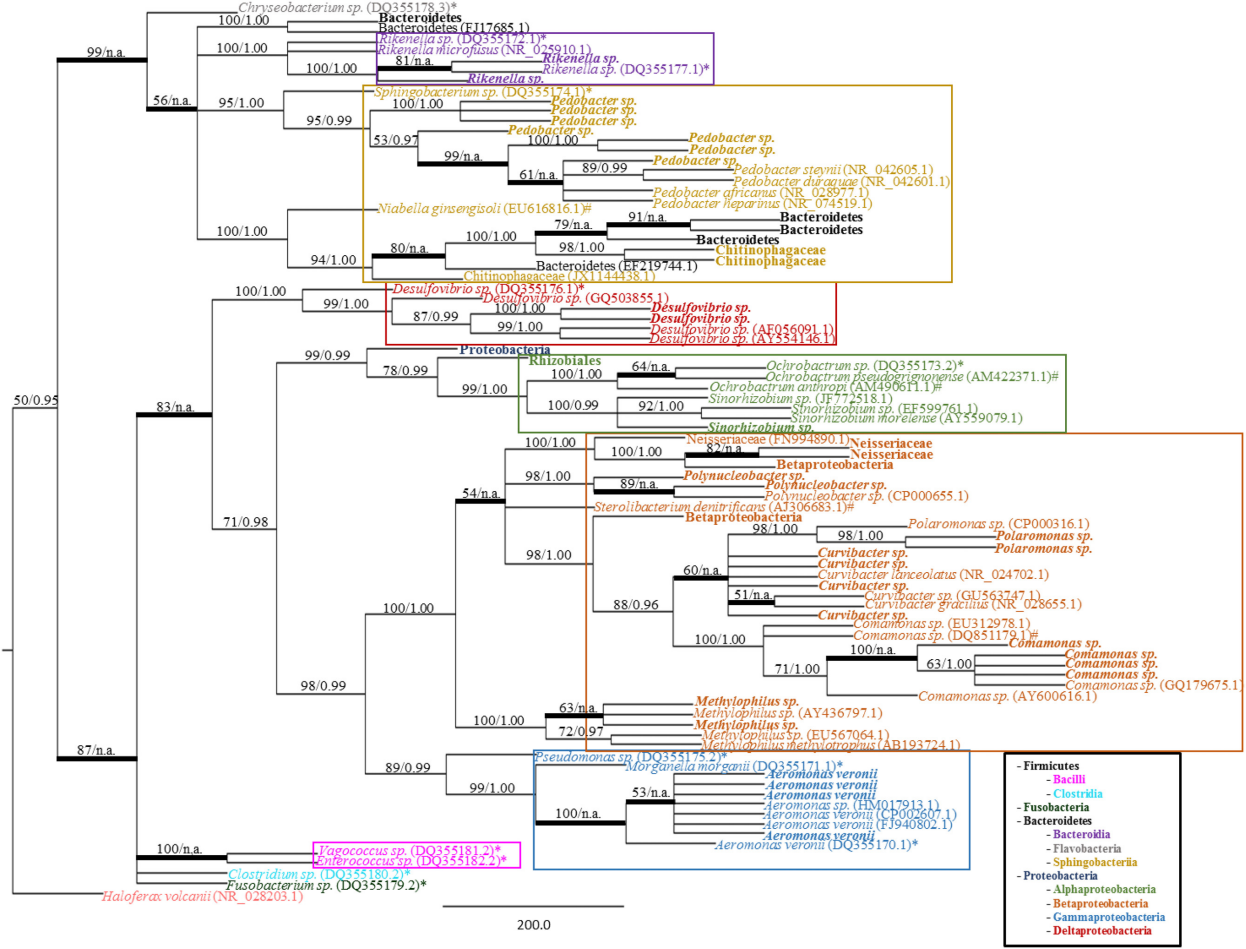
**Bacterial phylogeny from shed leech mucus**. Molecular phylogenetic tree of 16S rRNA gene sequences amplified from adult *H. verbana* mucosal secretions. A MP analysis tree created from approximately 900 aligned nucleotides is shown. Significance values, represented in MP bootstrap and Bayesian PP (BS/PP), are indicated at respective nodes. A bolded branch with “BS/n.a.” significance value refers to a branch that was not statistically supported by Bayesian analysis. Branch lengths are measured in number of substitutions over the whole sequence. Representative 16S rRNA sequences obtained within shed mucus are in bold, with other sequences obtained from NCBI indicated by accession numbers. Previously described leech isolates obtained through study of the gut and bladder systems are indicated by an ^*^ (Worthen et al., 2006) or # (Kikuchi et al., 2009), respectively. Color blocks indicate the Class housing the representative sequences.

A total of 140 clones from a mucosal 16S rRNA library were binned into RFLP types. A total of 25 unique RFLP types were identified (Table 1), sequenced, and their classification determined through BLASTn (Altschul et al., 1997). At least two clones from each RFLP type were sequenced in both directions with no sequence variation observed between clones. Phylogenetic reconstruction, using both MP and Bayesian analyses, confirmed the taxonomic identities of the retrieved sequences while also enabling the visualization of microbial diversity within shed mucus. The trees produced by both MP and Bayesian analyses were overall congruent (Figure 3). The 16S rRNA sequences retrieved from the mucosal clone library belonged to either Bacteroidetes or Proteobacteria, with 6 classes (i.e., Bacteroidia, Sphingobacteriia, Alphaproteobacteria, Betaproteobacteria, Gammaproteobacteria, and Deltaproteobacteria) represented within these phyla (Figure 3). A number of bacterial groups, including an unclassified Bacteroidetes, an unclassified Betaproteobacteria, *Pedobacter, Rikenella, Curvibacter*, and *Aeromonas veronii*, exhibited multiple RFLP types suggesting 16S rRNA nucleotide diversity within these taxa in leech-shed mucus (Table 1).

**Table 1:** **16S rRNA sequences obtained from adult medicinal leech, *H. verbana*, mucosal secretions**.

**Accession number**	**Bacterial division**	**Class**	**Order**	**Family**	**Genus**	**Species**	**No (%) of clones**
	Bacteroidetes^a^^b^^*^						9 (6.4%)
	Bacteroidetes	Bacteroidia	Bacteroidales	Rikenellaceae	*Rikenella/ Alistipes*^b^^*^		2 (1.4%)
		Sphingobacteriia	Sphingobacteriales	Chitinophagaceae			9 (6.4%)
				Sphingobacteriaceae	Pedobacter^**^		32 (23.0%)
	**Proteobacteria^b^**						1 (0.7%)
	Proteobacteria	Alphaproteobacteria	Rhizobiales				1 (0.7%)
				Rhizobiaceae	*Sinorhizobium/ Ensifer group*		1 (0.7%)
		Betaproteobacteria^a^^*^					5 (3.6%)
		Betaproteobacteria	Burkholderiales	Burkholderiaceae	*Polynucleobacter*		5 (3.6%)
				Comamonadaceae	*Polaromonas*		11 (7.9%)
					***Comamonas*^a^**		9 (6.4%)
					Curvibacter^*^		11 (7.9%)
			Methylophilales	Methylophilaceae	*Methylophilus*		5 (3.6%)
			Neisseriales	Neisseriaceae			3 (2.1%)
		Gammaproteobacteria	Aeromonadales	Aeromonadaceae	***Aeromonas***	*veronii*^b^^**^	31 (22.1%)
		Deltaproteobacteria	Desulfovibrionales	Desulfovibrionaceae	***Desulfovibrio*^b^**		5 (3.6%)

Isolates appearing in bold were previously described in 16S rRNA clone libraries of leech excretory

a (Kikuchi et al., 2009) and digestive

b *(Worthen et al., 2006) systems*;

*, ** *indicate two and three different RFLP types identified, respectively*.

The 16S rRNA mucosal clone library identified the presence of previously described leech symbionts. Specifically, the mucosal 16S rRNA clone library contained the sequences for; *A. veronii, Rikenella*-like bacteria and an unclassified Proteobacteria previously described within the GI tract; *Comamonas*, an unclassified Betaproteobacteria, and *Desulfovibrio* previously identified within the bladder; and an unclassified Bacteroidetes which has been described in both the leech digestive and excretory systems. Additionally, novel (i.e., not previously associated with *H. verbana*) sequences recovered were related to other Bacteroidetes (i.e., *Pedobacter* and a Chitinophagaceae family member) and Proteobacteria, specifically members of the Neisseriaceae family, and *Polynucleobacter, Polaromonas, Curvibacter, Sinorhizobium*, and *Methylophilus* genera. Further, the various RFLP types observed within certain taxa (i.e., the unclassified Bacteroidetes, *Pedobacter, Rikenella, Curvibacter*, unclassified Betaproteobacteria and *A. veronii*) are supported by the phylogenetic branching patterns of the corresponding sequences (Figure 3). For example, the *Rikenell*a-like bacterium found within shed mucus formed a monophyletic group with a *Rikenella*-like symbiont isolated from the leech digestive tract, with these forming a sister clade to an additional *Rikenella*-like symbiont isolate from the GI tract supporting diversity within this genus inside the leech host.

Additionally, while the closely related *Sphingobacterium* sp. symbionts have been isolated within the leech crop (Worthen et al., 2006) and bladders (Kikuchi et al., 2009), the positioning of this crop isolate in a sister clade to the mucosal *Pedobacter* isolates with strong statistical support (95% BS and 1.0 PP values), demonstrates their distinct identities (Figure 2 and Supplementary Figure S1). Comparisons of *Pedobacter* full-length 16S rRNA sequences generated by the Sanger-sequenced clone library resulted in identities ranging from 94.3 to 99.9%, indicating genetic diversity in *Pedobacter* isolates within mucus. Similar comparisons with the most closely related, previously characterized *Pedobacter* species (Supplementary Figure S2) revealed 16S rRNA sequence identities ranging from 93.9 to 98.6%, suggesting that mucosal isolates are housed within the *Pedobacter* genus, but may be distinct from known species.

**Figure 4:**
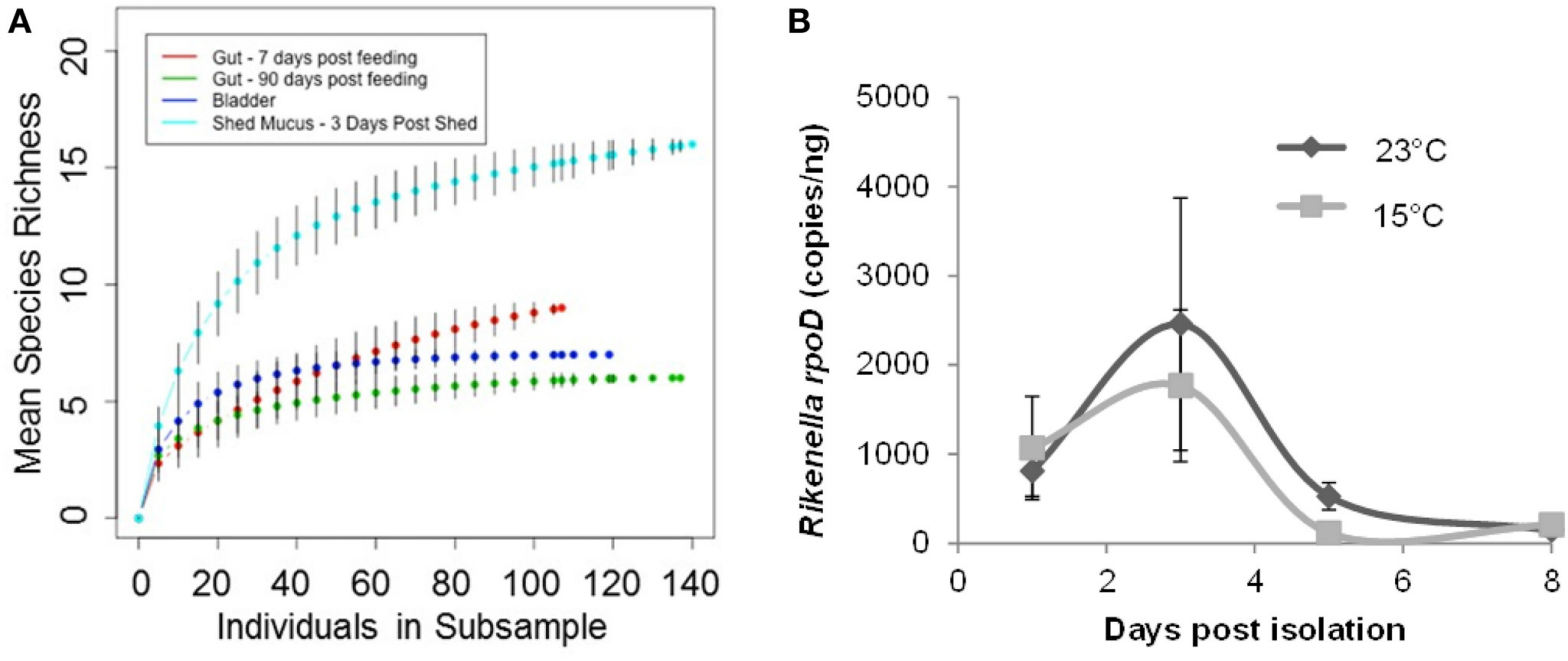
**Leech mucosal secretions contain a high diversity of microbial symbionts, to include the *Rikenella*-like bacterium. (A)** Rarefaction curves were generated using Hurlbert's formulation (Hurlbert, 1971) and are plotted with the mean species richness (y-axis) as a function of the number of individuals in each subsample (x-axis). The asymptotes rise less steeply as an increasing proportion of OTUs have been encountered. Error bars represent standard error. **(B)**. *Rikenella* density within mucus increases from Day 1 to 3, decreasing thereafter, mirroring the *A. veronii* population dynamics (Ott et al., 2014). *Rikenella* density was measured via qPCR using the *rpoD* gene. No significant differences were observed either between the two temperature regimens (*p* = 0.6868) or as the mucus aged (*p* = 0.0518, one-way ANOVA, *n* = 39).

Mean species richness, as determined through the comparison of Shannon-Weaver diversity index values, was higher in the mucosal clone library (2.34) relative to the bladder (1.4) and digestive tract clone libraries (1.11 and 1.38 at 7 d and 90 d following feeding; respectively) (Table 2). In further support, the higher asymptote obtained with the shed mucosal clone library rarefaction curve (Figure 4A) also supports higher species richness (Gotelli and Colwell, 2011) within shed mucus relative to the leech digestive tract and bladder microbial communities. However, the characterization of the microbiota within shed mucus appears to be incomplete through RFLP typing of the 16S rRNA clone library, as demonstrated when comparing observed richness values (Shannon-Weaver diversity indices) that account for only 10-23% of nonparametric estimators of expected richness (ACE, ICE, Chao 1 and Chao 2 estimators). This indicates the significant presence of low abundance sequences and unevenness in the abundance of taxa, as better captured with Illumina deep sequencing.

**Table 2:** **Species richness and coverage estimation of 16S rRNA clone libraries generated from *H. verbana* microbial niches**.

**Richness estimation**					
**Sample identity**	**Chao1**	**Chao2**	**ACE**	**ICE**	**Shannon-Weaver**
Shed Mucus-3 days post shed	20.5	20.5	16.69	16.69	2.341
Gut-7 days post feeding^a^	11.25	11.25	11.1	11.1	1.112
Gut-90 days post feeding^a^	6	6	6	6	1.377
Bladder^b^	7	7	7	7	1.398

Isolates previously described in

a *(Worthen et al., 2006) richness estimation was calculated for combined crop and intestinum data*;

b *(Kikuchi et al., 2009)*.

### *Rikenella*-population dynamics

Following the detection of the *Rikenella*-like bacteria also within shed mucus, we aimed to determine whether this symbiont may also be found proliferating within this substrate. We assayed the population density of *Rikenella*-like bacteria using quantitative PCR of the *rpoD* gene from mucus samples at two biologically relevant temperatures representing a pond's edge in *H. verbana*'s natural geographic distribution (Utevsky et al., 2010), the site of leech mating during the summer months (23°C, Sawyer, 1986), and a pond's base (15°C). No significant differences in *Rikenella* load were observed between the two temperature regimens (*p* = 0.6868, one-way ANOVA, *n* = 39). The *Rikenella*-like symbiont population dynamics (Figure 4B) mirrored those of *A. veronii* (Ott et al., 2014), reaching maximum abundance 3 days post shedding at both temperatures. A trend was observed in the *Rikenella*-load when examining time points following shedding (*p* = 0.0518, one-way ANOVA, *n* = 39), with Day 3 showing a higher abundance than Day 8 at both examined temperatures.

## Discussion

Many animals harbor mutualistic microbes that provide adaptive advantages to their hosts, often enabling unique lifestyles and habitation within restricted environments (reviewed in Sachs et al., 2004). These mutualists can be acquired through vertical or environmental transmission, although a mixture of both routes may occur (Ebert, 2013). With the medicinal leech, acquisition of a predominant gut symbiont, *A. veronii*, may occur as larvae during cocoon development (Rio et al., 2009) and following eclosion through contact with conspecific mucosal sheds (Ott et al., 2014).

Previous research has examined the leech gut microbiota utilizing both Sanger-sequenced 16S rRNA clone libraries (Worthen et al., 2006) and next-generation deep sequencing (Maltz et al., 2014), with the latter showing higher GI tract microbial diversity. In this study, we aimed to understand the taxonomic composition and relative abundance of the microbial community harbored within leech mucosal secretions and elucidate on their potential functional roles. Both independent culture-free molecular techniques, RFLP typing and Sanger sequencing of a 16S rRNA clone library and Illumina sequencing of the 16S rRNA V3-V4 hypervariable region, validated that shed mucus harbored bacteria from the Bacteroidetes and Proteobacteria phyla. The microbiota of leech mucosal secretions also consist of the previously described GI and bladder symbionts *Comamonas* sp., *Desulfovibrio* sp., and *Bdellovibrio* sp., as well as predominant digestive tract symbionts (*A. veronii* and *Rikenella*-like bacteria), in addition to a number of bacteria that have not been previously associated with the leech. The identification of these novel bacteria in association with shed mucosal casts supports the presence of yet to be described microbiotas within leeches, such as a mouth, pharyngeal and/or epidermal community. Additionally, microbial richness (i.e., the number of different types of bacteria characterized) was higher within the mucosal 16S rRNA clone library relative to the digestive and excretory tract clone libraries (Worthen et al., 2006; Kikuchi et al., 2009), further suggesting the presence of unexplored microbial communities within the leech. Interestingly, observed richness values (Shannon–Weaver diversity indices) account for only a fraction (i.e., 11-14%) of expected richness estimators, indicating the existence of a large proportion of low abundance sequences that were not discovered in the mucosal 16S rRNA clone library. This hypothesis is supported by the numerous genera uncovered in low abundance through Illumina deep-sequencing of the 16S rRNA V3-V4 hypervariable region.

In contrast to the 16S rRNA clone library, Illumina deep-sequencing identified a larger percentage of Bacteroidetes OTUs in comparison to the Proteobacteria (68 vs. 29%; respectively), while *Aeromonas* consisted of less than 1% of relative OTU abundance. The low number of *Aeromonas* sequences in our Illumina data set is not entirely surprising, as others have also encountered difficulties detecting *Aeromonas* during taxonomic assignment using only two variable regions (V4-V5) (Nelson et al., 2014). As Illumina is currently limited to short-read lengths, only small segments of the 16S rRNA gene can be characterized and it appears that, at least in regards to *Aeromonas*, longer sequences may be appropriate for proper taxonomic designation. Additionally, biases in OTU detection could also result from the use of different primers (Kautz et al., 2013) and primer efficiency, as well as in the initial amplification steps [i.e., PCR bias (Shendure and Ji, 2008)]. The generation of the Sanger-sequenced clone library was also dependent on RFLP typing through the use of the *HaeIII* restriction enzyme. If different taxa have similar RFLP banding patterns, these would have been placed in the same group. Although we attempted to reduce this occurrence by sequencing multiple clones from each haplotype, it is possible that some unique sequences were not detected. In congruence with our results, a study conducted on the microbiome of *Cephalotes* ants, comparing diversity obtained through 454 pyrosequencing to Sanger-sequenced clone libraries, resulted in similar discrepancies (Kautz et al., 2013). This study revealed that at higher taxonomic ranks the groups were qualitatively similar, although the proportions of each varied (Kautz et al., 2013).

The most abundant microbial group present within shed mucus, as determined by both sequencing methodologies, belonged to the *Pedobacter* genus, a novel leech associated bacterial group. Here, it is important to note that the abundance of certain bacteria within shed mucus may not be reflective of density within the leech host as the mucosal substrate may enable the proliferation, or contrastingly the decrease, of certain microbial groups over others. Within mucus, *Pedobacter* sequences are diverse, as exemplified by multiple RFLP banding patterns, comparisons of sequence identity, and when mucosal sequences are placed in *Pedobacter*-focused phylogenetic trees. The genus *Pedobacter* was first described by Steyn et al. (1998) to include heparinase-producing, obligate aerobic, Gram-negative rods discovered in soils and activated sludge. However, members of this genus have since been found in a wide array of environments, to include: freshwater lake sediment (An et al., 2009) and natural (Chun et al., 2014) and man-made water sources (Joung et al., 2010). The capabilities of most *Pedobacter* sp. are not well described, although a characteristic function, degradation of the anticoagulant heparin through the production of heparinase (Payza and Korn, 1956; Steyn et al., 1998), is of particular interest in regards to the ecology of the leech host. The role of heparinase in a putative symbiont of a blood-feeding host is unknown, but may facilitate feeding in conjunction with hirudin, a well-described anticoagulant produced by the leech salivary glands (Haycraft, 1884, 1894; Jacoby, 1904; Markwardt, 2002).

In addition to the highly abundant *Pedobacter*, a number of other bacteria were discovered within the mucus. We can only speculate as to their function in regards to the mucosal niche, however, previous studies on these lineages may help elucidate their roles. For example, the aerotolerant *Desulfovibrio* sp. are typically found in aquatic environments rich in organic materials and contribute toward the reduction of sulfate into sulfide (Adams and Postgate, 1959). Interestingly, members of the Comamonadaceae family are also capable of both sulfate cycling (Schmalenberger et al., 2008) (suggesting the presence of sulfate in the mucosal environment), in addition to denitrification in aqueous environments (Khan et al., 2002). Members of the Methylophilaceae family are also involved in denitrification (Kalyuhznaya et al., 2009). The functional roles of both Comamonadaceae and Methylophilaceae families indicate a potentially high level of denitrification activity within leech shed mucus. As the mucus is sloughed from the anterior to posterior end of the leech (Ott et al., 2014), and the leech nephridial system harbors 17 pairs of nephridia and bladders that empty their contents along the entire length of the leech (Sawyer, 1986), the mucus could contain significant levels of nitrogen and sulfate from leech waste (Dev, 1964; Nicholls and Kuffler, 1964; Zerbst-Boroffka, 1970; Sawyer, 1986), encouraging the growth of these denitrifiers.

A number of bacteria described within our leech-exuded mucus samples have recently been implicated as frequently occurring DNA contaminants of microbiomes due to reagent and laboratory contamination (Salter et al., 2014). These microbial contaminants prove to be a significant problem particularly within low biomass (Salter et al., 2014), i.e., 10^3^ or 10^4^ CFU/mL, and low quality/dilute (Lusk, 2014) samples where they tend to eclipse the true microbial community. However, the leech mucosal microbiota consists of a greater biomass than this critical threshold, with culturable microbes estimated to be at least 10^4^ CFU/mL, which falls short of accounting for unculturable microbes. Furthermore, by streaking mucus on various nutrient agar plates using traditional culturing approaches, a number of low abundance bacteria (i.e., each comprising <2% of total Illumina reads) were isolated, including *Aeromonas, Morganella, Stenotrophomonas, Delftia, Chryseobacterium*, and *Variovorax* species, verifying their presence and viability within mucus. In addition, the presence and proliferation of both *Aeromonas* and *Rikenella*-like bacteria within exuded mucus have been confirmed using qPCR (Ott et al., 2014, and current results), independent of Illumina amplicon generation and sequencing.

Interestingly, our most common mucosal community member, *Pedobacter*, is also recognized as a high frequency contaminant (Salter et al., 2014). However, we have determined that *Pedobacter* is not universally present within all leech-related samples, e.g., leech epithelial swabs, although the same DNA isolation buffers and procedures are used in sample processing. Additionally, preliminary metatranscriptome analyses of the exuded mucosal microbiome show that *Pedobacter* contributes a significant proportion of transcriptional activity within this environment (Ott, pers. obs.). These results support our conclusion that *Pedobacter* are likely not resulting from contamination but are bona fide community members of shed mucus.

Following the identification of a second predominant gut symbiont, *Rikenella*-like bacteria, also within mucus, its population dynamics were examined. As the dynamics between *A. veronii* and the *Rikenella*-like bacteria have been examined extensively within the leech host (Kikuchi et al., 2009; Bomar et al., 2011), we were interested to see if the relationship between these two symbionts may persist beyond the gut environment. We examined the growth of the *Rikenella*-like bacteria over a period of 8 days and we showed that the population dynamics mirrored those of *A. veronii* within shed mucus (Ott et al., 2014), suggesting a continuation of their syntrophic activities into this environment. As *Rikenella*-like bacteria in the crop are known to degrade host mucin glycans to short chain fatty acids, which serve as a carbon source for *A. veronii* (Bomar et al., 2011), it is not difficult to extrapolate this functional role to the glucosaminoglycan-rich mucosal environment. Therefore, *Rikenella*-like bacteria may serve a similar role in the maintenance and proliferation of mucosal *A. veronii* as in the gut. In return, it is possible that *A. veronii* ensures a habitable environment for the obligately anaerobic *Rikenella*-like bacteria through the removal of oxygen, indicating a relationship beyond one based solely on nutrition. Mucosal seeding through the digestive tract (Ott et al., 2014) and the eventual decrease in population may also occur in tandem, with symbionts either dispersing or dying after approximately 3 days post shedding, likely due to the exhaustion of resources.

The microbial community of leech mucus is diverse and harbors a number of novel bacteria that have not been previously associated with the leech. The discovery of this rich microbial community within mucus not only raises questions involving microbial interactions that may occur within this environment, but also whether these other bacteria utilize mucus as a transmission substrate for the infection of novel hosts. As many members of a host microbiota are thought to be transmitted as assemblages (Dominguez-Bello et al., 2010; Ebert, 2013; Makino et al., 2013; Aagaard et al., 2014), future work will explore the function of mucus as a symbiont mixing vessel by identifying loci which may be under different modes of selection (i.e., diversifying versus purifying) and the possibility of gene swapping in this environment. It is feasible that the mucosal environment provides symbionts with the opportunity to transmit to novel hosts as well as increase/mix their genetic repertoire, potentially enhancing their ability to adapt to changing environments.

## Author contributions

Conceived and designed the experiments: Brittany M. Ott, Rita V. M. Rio. Performed the experiments: Brittany M. Ott, Allen Rickards. Analyzed the data: Brittany M. Ott, Allen Rickards, Lauren Gehrke, Rita V. M. Rio. Contributed the reagents/material/analysis tools: Rita V. M. Rio. Wrote the paper: Brittany M. Ott, Rita V. M. Rio. Reviewed the paper Brittany M. Ott, Allen Rickards, Lauren Gehrke, Rita V. M. Rio.

### Conflict of interest statement

The authors declare that the research was conducted in the absence of any commercial or financial relationships that could be construed as a potential conflict of interest.

## References

[B1] Aagaard K. Ma J. Antony K. M. Ganu R. Petrosino J. Versalovic J. (2014). The placenta harbors a unique microbiome. Sci. Transl. Med. 6, 237ra65. 10.1126/scitranslmed.3008599 24848255 PMC4929217

[B2] Adams M. E. Postgate J. R. (1959). A new sulphate-reducing vibrio. J. Gen. Microbiol. 20, 252–257. 10.1099/00221287-20-2-252 13654719

[B3] Altschul S. F. Madden T. L. Schaffer A. A. Zhang J. Zhang Z. Miller W.. (1997). Gapped BLAST and PSI-BLAST: a new generation of protein database search programs. Nucleic Acids Res. 25, 3389–3402. 10.1093/nar/25.17.3389 9254694 PMC146917

[B4] An D. S. Kim S. G. Ten L. N. Cho C. H. (2009). *Pedobacter daechungensis* sp. nov., from freshwater lake sediment in South Korea. Int. J. Syst. Evol. Microbiol. 59(Pt 1), 69–72. 10.1099/ijs.0.001529-0 19126726

[B5] Bomar L. Maltz M. Colston S. Graf J. (2011). Directed culturing of microorganisms using metatranscriptomics. MBio 2, e00012–e00011. 10.1128/mBio.00012-11 21467263 PMC3069634

[B6] Chun J. Kang J. Y. Jahng K. Y. (2014). *Pedobacter pituitosus* sp. nov., isolated from Wibong falls. Int. J. Syst. Evol. Microbiol. 64, 3838–3843. 10.1099/ijs.0.065235-0 25168612

[B7] Dev B. (1964). Excretion and Osmoregulation in the Leech, *Hirudinaria Granulosa* (Savigny). Nature 202, 414. 10.1038/202414b0 14152849

[B8] Dominguez-Bello M. G. Costello E. K. Contreras M. Magris M. Hidalgo G. Fierer N.. (2010). Delivery mode shapes the acquisition and structure of the initial microbiota across multiple body habitats in newborns. Proc. Natl. Acad. Sci. U.S.A. 107, 11971–11975. 10.1073/pnas.1002601107 20566857 PMC2900693

[B9] Ebert D. (2013). The epidemiology and evolution of symbionts with mixed-mode transmission. Annu. Rev. Ecol. Evol. Syst. 44, 623–643 10.1146/annurev-ecolsys-032513-100555

[B10] Felsenstein J. (1985). Confidence limits on phylogenies: an approach using the bootstrap. Evolution 39, 783–791 10.2307/2408678 28561359

[B11] Fraune S. Augustin R. Anton-Erxleben F. Wittlieb J. Gelhaus C. Klimovich V. B.. (2010). In an early branching metazoan, bacterial colonization of the embryo is controlled by maternal antimicrobial peptides. Proc. Natl. Acad. Sci. U.S.A. 107, 18067–18072. 10.1073/pnas.1008573107 20921390 PMC2964230

[B12] Gotelli N. J. Colwell R. K. (2011). Estimating species richness, in Frontiers in Measuring Biodiversity, eds Magurran A. E. McGill B. J. (New York, NY: Oxford University Press), 39–54.

[B13] Haycraft J. B. (1884). On the action of a secretion obtained from the medicinal leech on the coagulation of the blood. Proc. R. Soc. B., 36, 478–487 10.1098/rspl.1883.0135

[B14] Haycraft J. B. (1894). Über die Einwirkung eines Sekretes des officiellen Blutegels auf die Gerinnbarkeit des Bluts. Naunyn Schmiedebergs Arch. Exp. Pathol. Pharmacol. 18, 209–217 10.1007/BF01833843

[B15] Holmes D. S. Bonner J. (1973). Preparation, molecular weight, base composition, and secondary structure of giant nuclear ribonucleic acid. Biochemistry 12, 2330–2338. 10.1021/bi00736a023 4710584

[B16] Hurlbert S. H. (1971). The nonconcept of species diversity: a critique and alternative parameters. Ecology 52, 577–586 10.2307/1934145 28973811

[B17] Jacoby Y. (1904). Über Hirudin. Dtsch Med. Wochenschr. 30, 786–787.

[B18] Joung Y. Kim H. Joh K. (2010). *Pedobacter yonginense* sp. nov., isolated from a mesotrophic artificial Lake in Korea. J. Microbiol. 48, 536–540. 10.1007/s12275-010-0010-4 20799098

[B18a] Jukes T. H. Cantor C. R. (1969). Evolution of Protein Molecules. New York, NY: Academic Press, 21–132.

[B19] Kalyuhznaya M. G. Martens-Habbena W. Wang T. Hackett M. Stolyar S. M. Stahl D. A.. (2009). Methylophilaceae link methanol oxidation to denitrification in freshwater lake sediment as suggested by stable isotope probing and pure culture analysis. Environ. Microbiol. Rep. 1, 385–392. 10.1111/j.1758-2229.2009.00046.x 23765891

[B20] Kautz S. Rubin B. E. Russell J. A. Moreau C. S. (2013). Surveying the microbiome of ants: comparing 454 pyrosequencing with traditional methods to uncover bacterial diversity. Appl. Environ. Microbiol. 79, 525–534. 10.1128/AEM.03107-12 23124239 PMC3553759

[B21] Khan S. T. Horiba Y. Yamamoto M. Hiraishi A. (2002). Members of the family Comamonadaceae as primary poly(3-hydroxybutyrate-co-3-hydroxyvalerate)-degrading denitrifiers in activated sludge as revealed by a polyphasic approach. Appl. Environ. Microbiol. 68, 3206–3214. 10.1128/AEM.68.7.3206-3214.2002 12088996 PMC126756

[B22] Kikuchi Y. Bomar L. Graf J. (2009). Stratified bacterial community in the bladder of the medicinal leech, *Hirudo verbana*. Environ. Microbiol. 11, 2758–2770. 10.1111/j.1462-2920.2009.02004.x 19678832

[B23] Kikuchi Y. Graf J. (2007). Spatial and temporal population dynamics of a naturally occurring two-species microbial community inside the digestive tract of the medicinal leech. Appl. Environ. Microbiol. 73, 1984–1991. 10.1128/AEM.01833-06 17277211 PMC1828818

[B24] Klindworth A. Pruesse E. Schweer T. Peplies J. Quast C. Horn M.. (2013). Evaluation of general 16S ribosomal RNA gene PCR primers for classical and next-generation sequencing-based diversity studies. Nucleic Acids Res. 41, e1. 10.1093/nar/gks808 22933715 PMC3592464

[B25] Kozich J. J. Westcott S. L. Baxter N. T. Highlander S. K. Schloss P. D. (2013). Development of a dual-index sequencing strategy and curation pipeline for analyzing amplicon sequence data on the MiSeq Illumina sequencing platform. Appl. Environ. Microbiol. 79, 5112–5120. 10.1128/AEM.01043-13 23793624 PMC3753973

[B26] Krediet C. J. Ritchie K. B. Cohen M. Lipp E. K. Sutherland K. P.. (2009). Utilization of mucus from the coral *Acropora palmata* by the pathogen *Serratia marcescens* and by environmental and coral commensal bacteria. Appl. Environ. Microbiol. 75, 3851–3858. 10.1128/AEM.00457-09 19395569 PMC2698349

[B27] Lane D. J. (1990). 16S/23S rRNA sequencing, in Nucleic Acid Techniques in Bacterial Systematics, ed Goodfellow E. S. M. (Chichester: John, Wiley and Sons), 115–175.

[B28] Larget B. Simon D. L. (1999). Markov chain Monte Carlo algorithms for the Bayesian analysis of phylogenetic trees. Mol. Biol. Evol. 16, 750–759 10.1093/oxfordjournals.molbev.a026160

[B29] Lusk R. W. (2014). Diverse and widespread contamination evident in the unmapped depths of high throughput sequencing data. PLoS ONE 9:e110808. 10.1371/journal.pone.0110808 25354084 PMC4213012

[B30] Makino H. Kushiro A. Ishikawa E. Kubota H. Gawad A. Sakai T.. (2013). Mother-to-infant transmission of intestinal bifidobacterial strains has an impact on the early development of vaginally delivered infant's microbiota. PLoS ONE 8:e78331. 10.1371/journal.pone.0078331 24244304 PMC3828338

[B31] Maltz M. A. Bomar L. Lapierre P. Morrison H. G. McClure E. A. Sogin M. L.. (2014). Metagenomic analysis of the medicinal leech gut microbiota. Front. Microbiol. 5:151. 10.3389/fmicb.2014.00151 24860552 PMC4029005

[B32] Markwardt F. (2002). Hirudin as alternative anticoagulant–a historical review. Semin. Thromb. Hemost. 28, 405–414. 10.1055/s-2002-35292 12420235

[B33] Michalsen A. Roth M. Dobos G. (2007). Medicinal Leech Therapy. New York, NY Thieme Medical Publishers.

[B34] Nelson M. C. Morrison H. G. Benjamino J. Grim S. L. Graf J. (2014). Analysis, optimization and verification of Illumina-generated 16S rRNA gene amplicon surveys. PLoS ONE 9:e94249. 10.1371/journal.pone.0094249 24722003 PMC3983156

[B35] Nicholls J. G. Kuffler S. W. (1964). Extracellular space as a pathway for exchange between blood and neurons in the central nervous system of the leech: ionic composition of glial cells and neurons. J. Neurophysiol. 27, 645–671. 14194964 10.1152/jn.1964.27.4.645

[B36] Nyholm S. V. Stabb E. V. Ruby E. G. McFall-Ngai M. J. (2000). Establishment of an animal-bacterial association: recruiting symbiotic vibrios from the environment. Proc. Natl. Acad. Sci. U.S.A. 97, 10231–10235. 10.1073/pnas.97.18.10231 10963683 PMC27829

[B37] Nylander J. A. A. (2004). MrModeltest v2. Evolutionary Biology, Centre, Uppsala, University, Uppsala.

[B38] Ott B. M. Cruciger M. Dacks A. M. Rio R. V. (2014). Hitchhiking of host biology by beneficial symbionts enhances transmission. Sci. Rep. 4, 5825. 10.1038/srep05825 25059557 PMC5376049

[B39] Payza A. N. Korn E. D. (1956). Bacterial degradation of heparin. Nature 177, 88–89. 10.1038/177088a0 13288602

[B40] Rio R. V. Maltz M. McCormick B. Reiss A. Graf J. (2009). Symbiont succession during embryonic development of the European medicinal leech, *Hirudo verbana*. Appl. Environ. Microbiol. 75, 6890–6895. 10.1128/AEM.01129-09 19648363 PMC2772434

[B41] Rohwer F. Seguritan V. Azam F. Knowlton N. (2002). Diversity and distribution of coral-associated bacteria. Mar. Ecol. Prog. Ser. 243, 1–10 10.3354/meps243001

[B42] Ronquist F. Huelsenbeck J. P. (2003). MrBayes 3: Bayesian phylogenetic inference under mixed models. Bioinformatics 19, 1572–1574. 10.1093/bioinformatics/btg180 12912839

[B43] Sachs J. L. Mueller U. G. Wilcox T. P. Bull J. J. (2004). The evolution of cooperation. Q. Rev. Biol. 79, 135–160 10.1086/383541 15232949

[B44] Salter S. J. Cox M. J. Turek E. M. Calus S. T. Cookson W. O. Moffatt M. F.. (2014). Reagent and laboratory contamination can critically impact sequence-based microbiome analyses. BMC Biol. 12:87. 10.1186/s12915-014-0087-z 25387460 PMC4228153

[B45] Sawyer R. T. (1986). Leech Biology and Behavior. Oxford: United Kingdom Clarendon Press

[B46] Schloss P. D. Handelsman J. (2005). Introducing DOTUR, a computer program for defining operational taxonomic units and estimating species richness. Appl. Environ. Microbiol. 71, 1501–1506. 10.1128/AEM.71.3.1501-1506.2005 15746353 PMC1065144

[B47] Schloss P. D. Westcott S. L. Ryabin T. Hall J. R. Hartmann M. Hollister E. B.. (2009). Introducing mothur: open-source, platform-independent, community-supported software for describing and comparing microbial communities. Appl. Environ. Microbiol. 75, 7537–7541. 10.1128/AEM.01541-09 19801464 PMC2786419

[B48] Schmalenberger A. Hodge S. Bryant A. Hawkesford M. J. Singh B. K.. (2008). The role of *Variovorax* and other Comamonadaceae in sulfur transformations by microbial wheat rhizosphere communities exposed to different sulfur fertilization regimes. Environ. Microbiol. 10, 1486–1500. 10.1111/j.1462-2920.2007.01564.x 18279342

[B49] Sekar R. Mills D. K. Remily E. R. Voss J. D.. (2006). Microbial communities in the surface mucopolysaccharide layer and the black band microbial mat of black band-diseased *Siderastrea siderea*. Appl. Environ. Microbiol. 72, 5963–5973. 10.1128/AEM.00843-06 16957217 PMC1563687

[B50] Shannon E. H. (1948). A mathematical theory of communication. Bell Syst. Tech. J. 27, 379–423; 623–656 10.1002/j.1538-7305.1948.tb00917.x

[B51] Sharon G. Rosenberg E. (2008). Bacterial growth on coral mucus. Curr. Microbiol. 56, 481–488. 10.1007/s00284-008-9100-5 18213480

[B52] Shendure J. Ji H. (2008). Next-generation DNA sequencing. Nat. Biotechnol. 26, 1135–1145. 10.1038/nbt1486 18846087

[B53] Shnit-Orland M. Kushmaro A. (2009). Coral mucus-associated bacteria: a possible first line of defense. FEMS Microbiol. Ecol. 67, 371–380. 10.1111/j.1574-6941.2008.00644.x 19161430

[B54] Steyn P. L. Segers P. Vancanneyt M. Sandra P. Kersters K. Joubert J. J. (1998). Classification of heparinolytic bacteria into a new genus, *Pedobacter*, comprising four species: *Pedobacter heparinus* comb. nov., *Pedobacter piscium* comb. nov., *Pedobacter africanus* sp. nov. and *Pedobacter saltans* sp. nov. proposal of the family Sphingobacteriaceae fam. nov. Int. J. Syst. Bacteriol. 48(Pt 1), 165–177. 10.1099/00207713-48-1-165 9542086

[B55] Swofford D. L. (2002). PAUP 4.0-Phylogenetic Analysis Using Parsimony. Version 4. Sunderland, MA: Sinauer Associates.

[B55a] Tamura K. Stecher G. Peterson D. Filipski A. Kumar S. (2013). MEGA6: molecular evolution genetics analysis version 6.0. Mol. Biol. Evol. 30, 2725–2729. 24132122 10.1093/molbev/mst197 PMC3840312

[B56] Thomas Y. Vogel G. Wunderli W. Suter P. Witschi M. Koch D.. (2008). Survival of influenza virus on banknotes. Appl. Environ. Microbiol. 74, 3002–3007. 10.1128/AEM.00076-08 18359825 PMC2394922

[B57] Utevsky S. Zagmajster M. Atemasov A. Zinenko O. Utevska O. Utevsky A. (2010). Distribution and status of medicinal leeches (genus *Hirudo*) in the Western Palaearctic: anthropogenic, ecological, or historical effects? Aquat. Conserv. 20, 198–210 10.1002/aqc.1071

[B58] Weisburg W. G. Barns S. M. Pelletier D. A. Lane D. J. (1991). 16S ribosomal DNA amplification for phylogenetic study. J. Bacteriol. 173, 697–703. 1987160 10.1128/jb.173.2.697-703.1991 PMC207061

[B59] Worthen P. L. Gode C. J. Graf J. (2006). Culture-independent characterization of the digestive-tract microbiota of the medicinal leech reveals a tripartite symbiosis. Appl. Environ. Microbiol. 72, 4775–4781. 10.1128/AEM.00356-06 16820471 PMC1489327

[B60] Zebe E. Roters F. J. Kaiping B. (1986). Metabolic changes in the medicinal leech *Hirudo medicinalis* following feeding. Comp. Biochem. Physiol. 84A, 49–55 10.1016/0300-9629(86)90041-1

[B61] Zerbst-Boroffka I. (1970). Organische Säurereste als wichtigste Anionen im Blut von *Hirudo medicinalis*. Z. Vergl. Physiol. 70, 313–321 10.1007/BF00297751

