# Supplementary material for: Characterization of shed medicinal leech mucus reveals a diverse microbiota
Source: Front Microbiol. 2015 Jan 9;5:757. doi: 10.3389/fmicb.2014.00757 (PMC4288373; doi:10.3389/fmicb.2014.00757)
Supplement: Supplementary file 1 [file Image1.PDF]

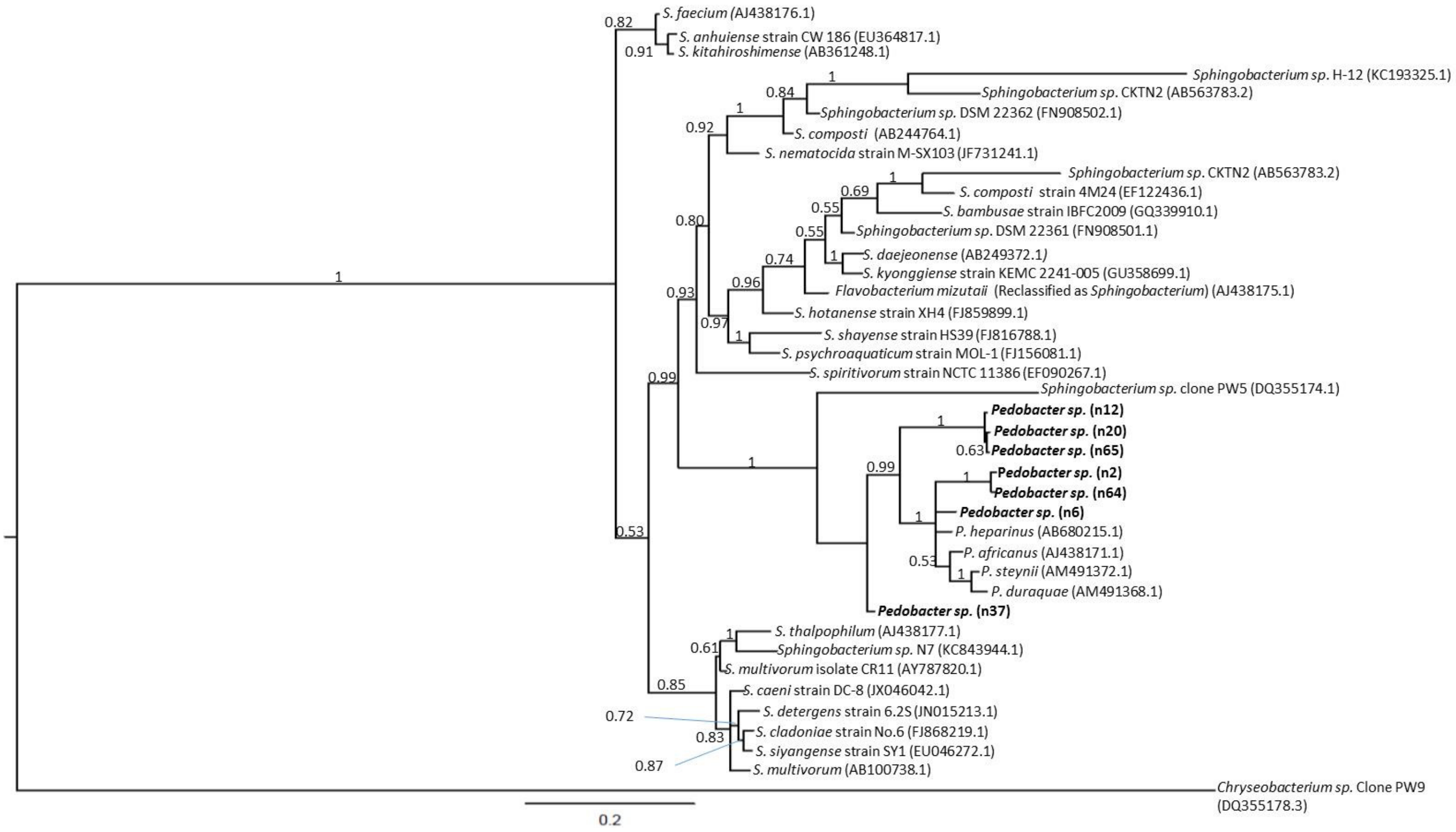

Supplementary Figure 1. Molecular 16S rRNA phylogenetic tree exhibiting the separation between the novel leech symbiont, *Pedobacter sp*., and its close relative, *Sphingobacterium sp*. A Bayesian analysis tree created from approximately 1400 aligned nucleotides is shown. Significance values, represented in Bayesian PP, are indicated at respective nodes. Branch lengths are measured in number of substitutions over the whole sequence. Representative *Pedobacter sp.* 16S rRNA sequences obtained within shed mucus are in bold, with other sequences obtained from NCBI indicated by accession numbers.
